# Acarbose Protects Glucolipotoxicity-Induced Diabetic Nephropathy by Inhibiting Ras Expression in High-Fat Diet-Fed db/db Mice

**DOI:** 10.3390/ijms232315312

**Published:** 2022-12-05

**Authors:** Tung-Wei Hung, Meng-Hsun Yu, Tsung-Yuan Yang, Mon-Yuan Yang, Jia-Yu Chen, Kuei-Chuan Chan, Chau-Jong Wang

**Affiliations:** 1 School of Medicine, Institute of Medicine, Chung Shan Medical University, Taichung 402, Taiwan; 2 Department of Medicine, Division of Nephrology, Chung Shan Medical University Hospital, Taichung 402, Taiwan; 3 Department of Nutrition, Chung Shan Medical University, No. 110, Sec. 1, Jianguo N. Road, Taichung 402, Taiwan; 4 Department of Health Industry Technology Management, Chung Shan Medical University, Taichung 402, Taiwan; 5 Department of Internal Medicine, Chung Shan Medical University Hospital, Taichung 402, Taiwan; 6 Department of Medical Research, Chung Shan Medical University Hospital, Taichung 402, Taiwan

**Keywords:** acarbose, db/db mice, diabetic nephropathy, Ras

## Abstract

Diabetic nephropathy (DN) exacerbates renal tissue damage and is a major cause of end-stage renal disease. Reactive oxygen species play a vital role in hyperglycemia-induced renal injury. This study examined whether the oral hypoglycemic drug acarbose (Ab) could attenuate the progression of DN in type 2 diabetes mellitus mice. In this study, 50 mg/kg body weight of Ab was administered to high-fat diet (HFD)-fed db/db mice. Their body weight was recorded every week, and the serum glucose concentration was monitored every 2 weeks. Following their euthanasia, the kidneys of mice were analyzed through hematoxylin and eosin, periodic acid Schiff, Masson’s trichrome, and immunohistochemistry (IHC) staining. The results revealed that Ab stabilized the plasma glucose and indirectly improved the insulin sensitivity and renal functional biomarkers in diabetic mice. In addition, diabetes-induced glomerular hypertrophy, the saccharide accumulation, and formation of collagen fiber were reduced in diabetic mice receiving Ab. Although the dosages of Ab cannot decrease the blood sugar in db/db mice, our results indicate that Ab alleviates glucolipotoxicity-induced DN by inhibiting kidney fibrosis-related proteins through the Ras/ERK pathway.

## 1. Introduction

Diabetes is a well-known risk factor for chronic kidney disease, and it exacerbates renal tissue damage. Diabetic nephropathy (DN) is a major complication of diabetes and remains a leading cause of end-stage renal disease in individuals with type 1 or 2 diabetes. The major structural characteristics of DN include proteinuria, the thickening of the glomerular basement membrane (GBM), and mesangial expansion due to the accumulation of extracellular matrix (ECM) proteins [1]. Pathophysiology is characterized by inflammation, fibrosis, and hypertrophy in renal cells caused by various cytokines and growth factors, such as the transforming growth factor β1, angiotensin II, and the platelet-derived growth factor [2]. The main modifiable risk factors for DN are hypertension, glycemic control, and dyslipidemia [3].

In the diabetic milieu, metabolic derangements and hemodynamic alterations, particularly the renin-angiotensin system activation [4], lead to efferent arteriolar vasoconstriction and the production of pro-inflammatory and pro-fibrotic molecules. Some of the newly discovered signaling pathways or molecules, including Notch, Wnt, mammalian target of rapamycin (mTOR), toll-like receptors (TLRs), and small GTPase, may modulate the regulation and expression of ECM in DN [5]. By targeting the relevant genes, small GTPases such as those from Rho, Ras, and Rab families may also influence the accumulation of ECM proteins and the development of renal fibrosis in hyperglycemic states [6]. The Ras family also plays an essential role in the accumulation of ECM and pathogenesis of DN [7]. Ras/Rho GTPases are stimulated in the diabetic milieu, such as advanced glycation end products (AGEs), reactive oxygen species, the hexosamine pathway, and oxidized low-density lipoproteins in renal cells [8].

Despite the identification of multiple pathogenic mechanisms in DN, limited success has been achieved in its medical treatment. Currently, there are no specific medical treatments to prevent fibrosis in DN [9].

α-Glucosidase inhibitors (AGIs) are a class of oral glucose-lowering drugs used exclusively for the treatment or prevention of type 2 diabetes mellitus. Three AGIs used in clinical practice are acarbose, voglibose, and miglitol [10]. Acarbose (Ab) is the main gradient of Glucobay, a clinical drug for the treatment of type 2 diabetes. This α-AGI is a pseudotetrasaccharide extracted from microorganisms. It blocks the action of the homonymous enzyme located in the small intestinal brush border, which is involved in the hydrolysis of oligosaccharides, trisaccharides, and disaccharides into glucose and other monosaccharides [11]. The selective inhibition of α-glucosidase decreases glucose production and delays its absorption, consequently reducing postprandial glucose variations without affecting insulin secretion and increasing the risk of hypoglycemia. Additionally, a treatment with AGIs, especially Ab, has beneficial effects on the lipid levels, blood pressure, coagulation factors, carotid intima-media thickness, and endothelial dysfunction [5]. Postprandial hyperglycemia is inversely associated with the time interval between diabetes and the development of DN in people with type 1 or 2 diabetes [12]. A study reportedly indicates that there was a significant decrease in the incidence and severity of glomerulosclerosis in the Cohen diabetic rat treated with Ab for 3 months [13]. Recently, Song et al. explored that Ab reduced low-grade albuminuria in type 2 diabetes [14]. These results imply that Ab potentiates the renal protective effect, but the underling mechanisms remain unknown. In this study, db/db+high-fat diet (HFD) mice were conducted to evaluate whether Ab can protect glucolipotoxicity-induced DN.

## 2. Results

### 2.1. Effects of acarbose on Blood Glucose Level and Body Weight of db/db Mice

**Figure 1:**
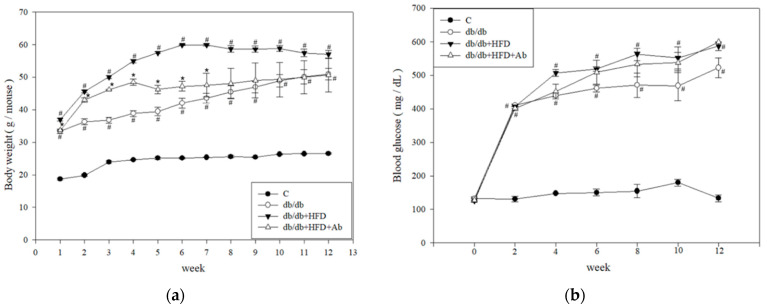
Ab decreased body weight and blood glucose in db/db mice with an HFD. During the experiment, db/db mice were fed an HFD and Ab. (**a**) The body weight of mice was measured once a week. (**b**) Fasting blood glucose from mice was tested every two weeks. Data are presented as mean ± SD; *n* = 3 mice per group. The results were analyzed using the analysis of variance. #, *p* < 0.05 compared with the control group. *, *p* < 0.05 compared with the db/db+HFD group.

To confirm the severity of diabetes in mice and the effect of acarbose (Ab) on mice, we measured their body weight every week and measured the fasting blood glucose level of mice every 2 weeks. The results revealed that the body weight of db/db mice and high-fat diet (HFD)-fed mice was significantly higher than that of control mice. However, the body weight of db/db+HFD+Ab mice was significantly lower than that of db/db+HFD mice at 1 to 7 weeks, but no significant difference was observed for week 8 (Figure 1A). Similarly, in HFD-fed mice, the blood glucose level raised continuously after 2 weeks and was significantly higher than in the control mice, whereas no significant difference was observed in the blood glucose level of db/db+HFD+Ab mice and db/db+HFD mice (Figure 1B). The results were in accordance with the inhibition of a polysaccharide degradation by Ab; nevertheless, Ab could not decrease the blood glucose in db/db mice with hyperglycemia in our current study. 

### 2.2. Effects of Acarbose on the Serum Biochemical Parameters of db/db Mice

We further explored the effects of Ab on diabetic mice (Table 1). HbA1c represents the average plasma glucose concentration for a 3-month period. The HbA1c level was high in the db/db and db/db+HFD groups, whereas that in the db/db+HFD+Ab group decreased slightly but was still higher than the standard value. The insulin levels or HOMA-IR increased significantly in the db/db and db/db+HFD groups, indicating a potential insulin resistance, and the insulin levels or HOMA-IR decreased after the Ab treatment. The blood lipid parameters, cholesterol, triglycerides, and LDL-C levels were significantly higher in the db/db+HFD group than in the control group and not in the db/db+HFD+Ab group. Although HDL-C related to a cholesterol clearance was the highest in the db/db+HFD group; the total cholesterol/HDL-C ratio was the highest in the db/db+HFD group.

Furthermore, the results revealed significantly higher levels of the kidney-related parameters of blood urea nitrogen (BUN), creatinine (Cre), and uric acid (UA) in the db/db+HFD group than in the control group, and a downward trend was observed after the Ab treatment. These results imply that Ab is effective in counteracting the HFD-induced kidney dysfunction. 

### 2.3. Effects of Acarbose on Renal Histological CHANGES in Experimental Animals

**Figure 2:**
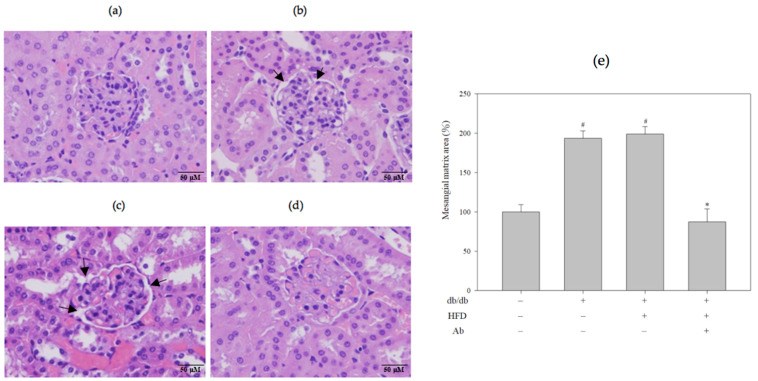
Ab decreased glomerular hypertrophy and expansion of the mesangial matrix in the glomeruli. Kidney slices were embedded in paraffin and stained with hematoxylin and eosin (H&E). Light photomicrographs of kidney sections from (**a**) control group (C57BL/6 mice), (**b**) db/db group, (**c**) db/db+HFD group, (**d**) db/db+HFD+Ab group were displayed (400× magnification). Arrow indicates the location of mesangial matrix expansion in the glomeruli. (**e**) Quantification of the mesangial matrix area. Fifteen microscopic fields of glomeruli per mice were randomly examined. Data are presented as mean ± SD; *n* = 3 mice per group. The results were analyzed using the analysis of Student’s *t*-test. #, *p* < 0.05 compared with the control group. *, *p* < 0.05 compared with the db/db+HFD group.

**Figure 3:**
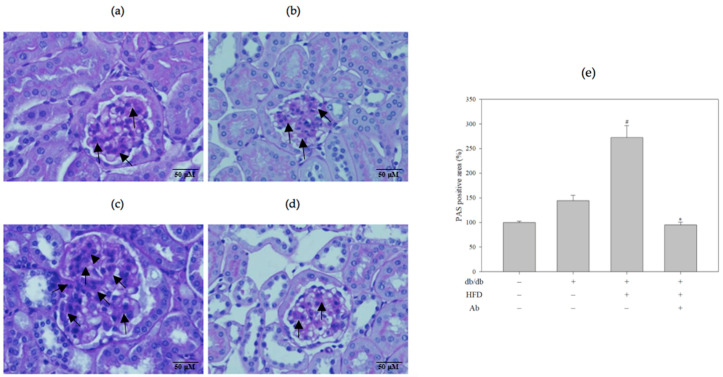
Ab decreased saccharide accumulation in the glomeruli. Kidney slices were embedded in paraffin and stained with PAS reagent. Light photomicrographs of kidney sections from (**a**) control group (C57BL/6 mice), (**b**) db/db group, (**c**) db/db+HFD group, (**d**) db/db+HFD+Ab group were displayed (400× magnification). The arrow indicates saccharide expression in the glomeruli. (**e**) Quantification of the PAS-positive area. Fifteen microscopic fields of glomeruli per mice were randomly examined. Data are presented as mean ± SD; *n* = 3 mice per group. The results were analyzed using the analysis of Student’s *t*-test. #, *p* < 0.05 compared with control group. *, *p* < 0.05 compared with the db/db +HFD group.

**Figure 4:**
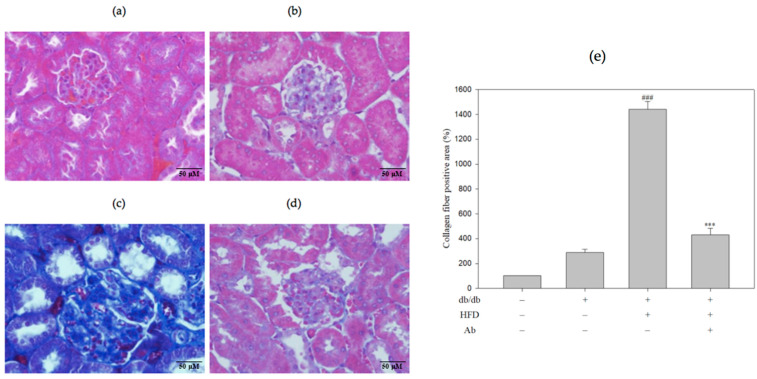
Ab reduced collagen fiber expansion in the kidneys. Kidney slices were embedded in paraffin and stained by Masson’s trichrome reagent. Light photomicrographs of kidney sections from (**a**) control group (C57BL/6 mice), (**b**) db/db group, (**c**) db/db+HFD group, (**d**) db/db+HFD+Ab group were displayed (400×magnification). The blue color on the renal biopsy represented the expression of collagen fiber. (**e**) Quantification of the collagen fiber-positive area. Data are presented as mean ± SD; *n* = 3 mice per group. The results were analyzed using Student’s *t*-test. ###, *p* < 0.001 compared with the control group. ***, *p* < 0.001 compared with the db/db+HFD group.

The renal sections of db/db+HFD mice exhibited glomerular lesions characterized by glomerular hypertrophy and the increased expansion of the mesangial matrix (increased Bowman’s capsule space) in the glomeruli by hematoxylin and eosin (H&E) staining (Figure 2). Figure 2E presents the quantification of the mesangial matrix area. In db/db+HFD+Ab mice, the general structure was rescued, and the Bowman’s capsule space was narrowed. In addition, PAS staining revealed that in the db/db+HFD group, the saccharide accumulation in glomeruli was significantly higher than in the control group and decreased after the Ab treatment (Figure 3, PAS staining). Masson’s trichrome stain was used to demonstrate renal fibrosis as a collagen fiber deposition (Figure 4) in the experimental groups. The db/db+HFD group exhibited marked increases in the PAS-stained positive areas, the amount of collagen, and increased fibrosis compared with the db/db+HFD+ Ab group.

### 2.4. Effects of acarbose Treatment on Renal Ras Expression in Diabetic Mice

**Figure 5:**
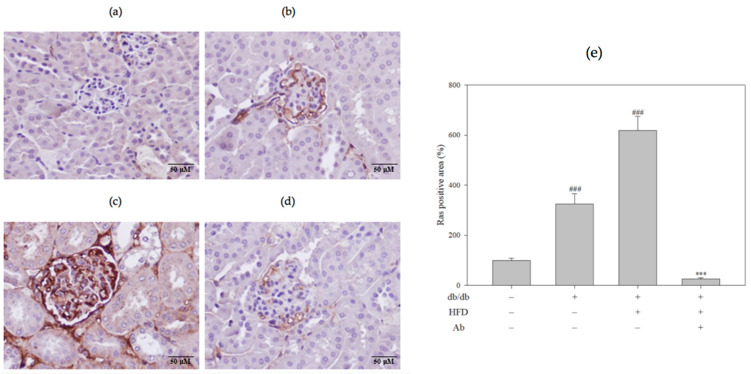
Ab decreased renal Ras expression. Renal Ras expression was detected with immunohistochemical analysis. Light photomicrographs of kidney sections from the (**a**) control group (C57BL/6 mice), (**b**) db/db group, (**c**) db/db+HFD group, (**d**) db/db+HFD+Ab group were displayed (400× magnification). The brown color on the renal biopsy represented Ras expression. (**e**) Quantification of the Ras-positive area. Data are presented as mean ± SD; *n* = 3 mice per group. The results were analyzed with Student’s *t*-test. ###, *p* < 0.001 compared with the control group. ***, *p* < 0.001 compared with the db/db+HFD group.

Ras family members are downstream convergence points expressed in renal injury and play a crucial role in the progression of diabetes and its complications [15]. This study showed that Ab can reduce saccharide and collagen accumulation in the histological sections of the kidneys and thus delays renal fibrosis. Therefore, this experiment explored whether Ab affects the renal Ras expression by performing immunohistochemistry (IHC) staining (Figure 5). The Ras expression in the db/db+HFD group was higher than in the control group. After the Ab treatment, the Ras expression decreased. Figure 5E presents the quantitative results.

### 2.5. Acarbose Inhibits Renal Fibrosis-Related Pathways in Mice

**Figure 6:**
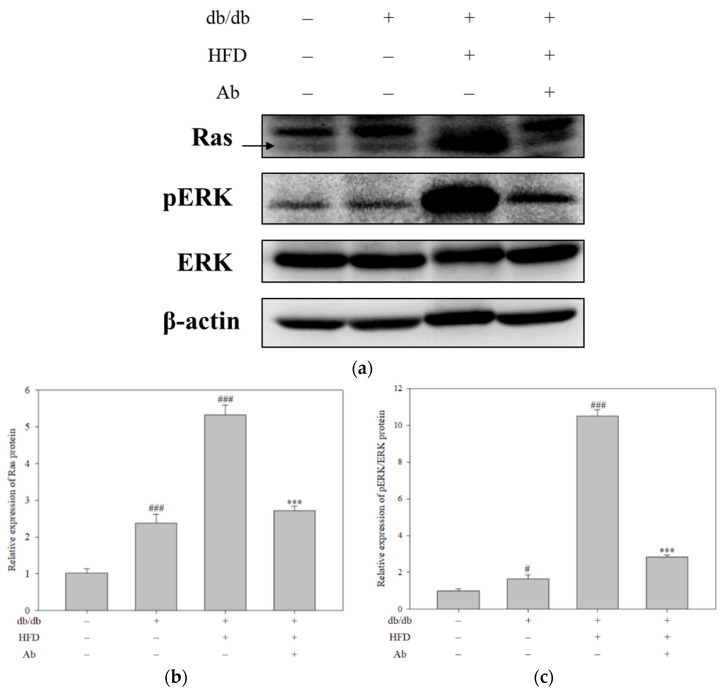
Ab decreased the expression of Ras-related proteins in the kidney. The expression of Ras-related protein in the kidney was detected through Western blotting. The renal tissue was derived from C57BL/6 mice, db/db mice, db/db mice with HFD, db/db mice with HFD, and Ab. (**a**) The results were analyzed using Image-Pro plus and displayed. Quantification of (**b**) Ras protein expression and (**c**) the pERK/ERK protein expression. β-actin was used as a loading control. Data are presented as mean ± SD from three samples for each group. The results were analyzed with Student’s *t*-test. *# p* < 0.05, ###, *p* < 0.001 compared with the control group. ***, *p* < 0.001 compared with the db/db+HFD group.

Ras phosphorylates ERK to cause renal fibrosis [16]. Because Ab reduced the renal Ras expression, we examined whether Ab affects the expression of the downstream proteins of Ras (Figure 6A). Western blotting indicated no difference in the expression of ERK, and the HFD induced a significant increase in the phosphorylation of ERK (pERK) (Figure 6A). The expression of pERK and Ras was higher in the db/db+HFD group than in the control group and decreased after the Ab treatment (Figure 6B,C). Together, the data indicate that Ab can reduce the accumulation of saccharide and fibrosis collagen in the kidneys of diabetic mice by inhibiting an ERK phosphorylation through the downregulation of the Ras expression.

## 3. Discussion

Diabetic nephropathy (DN) is the major cause of end-stage renal disease [2]. We demonstrated that acarbose (Ab) improved the glycemic control in high-fat diet (HFD)-induced type 2 diabetes mice and decreased the hyperglycemia-induced structural damage to the kidneys; these effects were associated with the inhibition of Ras-regulated renal fibrosis.

Mouse models are useful for identifying and testing novel therapeutic strategies that could translate into a better protection against human disease. The diabetogenic diet-fed C57BL/6 diabetic mice model has been widely used in DN research, and these mice are particularly susceptible to HFD-induced type 2 diabetes [17]. The HFD hyperinsulinemic db/db mice model displays a substantial glomerular pathology, including a mesangial matrix expansion and modest albuminuria [18]. In this study, an HFD was used to induce type 2 diabetes in db/db mice. As described previously, HFD-fed mice exhibited obesity, hyperglycemia, and an insulin resistance. Although, the db/db mice also exhibited significant hyperlipidemia, as well as a high body weight, blood urea nitrogen (BUN), creatinine (Cre), blood sugar, HbA1c, and uric acid (UA)compared with the control group. However, the characteristics of diabetic nephropathy, including glomerular atrophy, mesangial expansion, saccharide accumulation, and fibrosis, with the expansion of collagen fibers, were found in the kidney sections of the db/db+HFD group. The kidneys of type 2 diabetic mice contain a large amount of fat, which could further induce lipid peroxidation and oxidative stress, thus mediating the related downstream signals. DN including glomerular atrophy, mesangial matrix expansion, the accumulation of saccharide in the glomeruli, and collagen fiber expansion was found in the kidney sections of the db/db group, which is compatible with a previous study’s findings [15]. Notably, Ab attenuated the morphological alterations and improved the renal function, indicating that it could reduce the risk of renal dysfunction by preventing a renal structural and cellular injury.

Ab, an α-AGI, reduces the monosaccharide availability in the small intestine and therefore suppresses the postprandial blood glucose levels [19]. According to a previous study, Ab reduces the blood glucose and glycated hemoglobin (HbA1c) levels in patients with type 2 diabetes. The reductions occur without increases in the plasma insulin levels. Thus, Ab decreases insulin resistance [20]. In the present study, Ab improved the glycemic control and reduced cholesterol, triglyceride, and low-density lipoprotein levels in mildly diabetic mice without an insulin treatment.

Despite the relationship between the glycemic control and diabetes-induced kidney dysfunction, the effects of Ab therapy on diabetic renal complications remain less evaluated, especially in humans [21]. Most of the evidence is based on the findings observed in animal models. In a previous animal model, Ab attenuated renal dysfunction, including the accumulation of extracellular matrix (ECM), deposition of mesangial cell matrix, and glomerular basement membrane (GBM) thickening in the kidney sections [22]. In the animal model, Ab had renoprotective effects, particularly in those with or at risk of diabetes.

In this study, the blood glucose continued to rise in HFD-fed mice, and the levels were significantly higher than in the control group. Furthermore, no significant difference was observed in the blood glucose levels between the db/db+HFD+Ab and db/db+HFD groups. Ab, which belongs to the glucosidase inhibitor, can inhibit the degradation of the polysaccharide to glucose but it cannot lower the blood glucose in db/db mice with hyperglycemia. This study indicated that Ab attenuated morphological alterations and improved the renal function to reduce renal dysfunction. It implied that Ab decelerated DN via inhibiting glucolipotoxicity but not the glucose level.

Glomerular mesangial cells and interstitial fibroblasts are renal cells that play an essential role in the development of renal fibrosis [23]. The proliferation of renal fibroblasts has been implicated in the pathophysiology of interstitial fibrosis, because the activation leads to the myofibroblast phenotype and a subsequent increase in the ECM deposition [24]. Hyperglycemia and TGF-β are pathogenic factors for DN [25]. In addition, many of the cytokines, growth factors, and integrins involved in proliferation are known to activate the intracellular signaling pathways that converge on Ras proteins [26].

Hyperglycemia promotes the formation of AGEs. Interacting with their specific receptor, AGEs induce the generation of reactive oxygen species, mediate their action through oxidative stress, and accelerate lipid peroxidation [27]. Because the kidneys of type 2 diabetic rats contain a large amount of fat, they could further produce lipid peroxidation and oxidative stress, thus mediating the related downstream signals [28]. In hyperglycemia, Ras/Rho-kinase, an effector of small-GTPase binding proteins, is implicated in the pathogenesis of diabetic kidney disease by inducing endothelial dysfunction, podocyte abnormality, excessive ECM production, tubulointerstitial fibrosis, tubular atrophy, and mesangial sclerosis in glomeruli [29]. Ras GTPases are activated during renal fibrosis and play crucial roles in regulating both the cell proliferation and TGF-β-induced epithelial–mesenchymal transition [30]. Ras family members are downstream convergence points for signal cascades mediated by many cell surface receptors with the ligands expressed in renal injury, such as transforming the growth factor β (TGF-β), platelet-derived growth factor, angiotensin II, and epidermal growth factor, allowing for the transduction of the signal to the downstream pro-fibrotic effectors. In a renal fibrosis model, tubulointerstitial fibrosis increased the Ras, ERK, and Akt activation [31]. Ras protein is associated with DN. ERK, an MAPK, is an essential kinase in the intracellular signal transduction system leading to cell proliferation and ECM protein synthesis [32].

This study explored how Ab affects the renal Ras expression. The Ras expression in the db/db+HFD group was higher than that in the control group and decreased after an Ab treatment. A further analysis revealed that Ab attenuated morphological alterations such as glomerular atrophy, the mesangial matrix expansion in the glomeruli, decreased the accumulation of saccharide and collagen fiber in the glomeruli, and improved the renal function. To summarize, the Ab attenuated DN by inhibiting ERK through the Ras signaling pathway.

## 4. Materials and Methods

### 4.1. Preparation of Drug

Acarbose (Ab) was provided by Chung-Shan Medical University Hospital (Tanglu^®^ Tablets 50 mg; English name: Glucobay^®^ Tablets 50 mg, Bayer Schering Pharma, Taiwan). The license numbers are DOH Import No. 020786 and DOH Import No. 020787. Ab (tablets) was ground to the powder and added into distilled water until its use.

### 4.2. Animal Experiments

Six-week-old BKS.Cg-*Dock7*^m^+/+*Lepr*^db^/JNarl (db/db) and C57BL/6 male mice were obtained from the National Laboratory Animal Center (Taipei, Taiwan). The animal experiment was approved by the Animal Model Experimental Ethics Committee of Chung-Shan Medical University (IACUC, CSMU, approval no. 2433) and conformed to the recommendations in the Guide for the Care and Use of Laboratory Animals of the National Institutes of Health. The mice were acclimated to the laboratory conditions (22 ± 2 °C, 65 ± 5% relative humidity, and 12 h light/dark cycle). The db/db mice were acclimated to sterile independent ventilated breeding cages and were provided sterilized drinking water and litter. The experiment was conducted on a sterile operating table. The C57BL/6 mice were acclimated to general breeding cages and were provided with distilled water.

### 4.3. Animal Grouping and Drug Administration

After 1 week of adaptation, the mice were divided into four groups (*n* = 3 per group). Each group was fed for 14 weeks. Each mouse was housed in a cage under regular conditions (12 h light/dark cycle) with free access to water and food. The control group (C57/BL/6 mice) and one group of db/db mice were fed the same normal diet (LabDiet Laboratory Rodent Diet 5001). The remaining groups were fed an HFD diet (containing 15% lard oil and 0.5% cholesterol supplemented in diet) according to the manufacturer’s instructions and previous research [33]. The groups were (1) the control; (2) db/db; (3) db/db+HFD; and (4) db/db+HFD+Ab groups. Ab was administered by tube feeding at 50 mg/kg [34]. The fasting blood sugar level was measured every 2 weeks, and the body weight was measured every week. At the end of the 12-week experiment, all the mice were killed, and their kidneys and blood were harvested. The blood samples were centrifuged at 3000× *g* for 10 min at 4 °C and stored at −80 °C until analysis. The kidneys were immediately subjected to different processing methods for the histological, biochemical, and immunoblot analyses.

### 4.4. Serum Biochemical Measurements

The serum samples were collected into sodium heparin tubes and centrifuged at 3000× *g* rpm (1400 g) for 10 min at 4 °C. The concentrations of HbA1c, cholesterol, triglyceride, high-density lipoprotein cholesterol (HDL-C), low-density lipoprotein cholesterol (LDL-C), blood urea nitrogen (BUN), creatinine (Cre), and uric acid (UA) were measured using enzymatic colorimetric methods using commercial kits (Randox Laboratories, Antrim, UK). The insulin was measured with an ELISA kit (Mercodia AB, Uppsala, Sweden). 

### 4.5. Renal Morphology Assessment and Immunohistochemistry Analysis

Hematoxylin–eosin (H&E), periodic acid Schiff (PAS), and Masson’s trichrome staining were performed following the conventional histochemical methods described previously [35].

The kidney specimens were obtained, sectioned, and immediately fixed in 10% formol saline for 24 h. The paraffin blocks were prepared, and 5 µm thick sections were subjected to H&E and PAS staining. The histological analysis was performed using a Nikon Eclipse E600 microscopy system (Nikon Instruments, Melville, NY, USA) without knowledge of the identity of the various groups. A pathologist blinded to the study protocol coded and examined the slides to identify the histological alterations. The morphometry and statistical analysis of the mean width of Bowman’s space in H&E-stained sections were conducted in 10 nonoverlapping fields for each group.

### 4.6. Immunohistochemistry

The kidney specimens were fixed in 4% paraformaldehyde solution and embedded in paraffin. After the deparaffinization and rehydration, 5 mm kidney sections were treated with 3% H_2_O_2_ for 10 min and 1% bovine serum albumin in phosphate-buffered saline for 30 min. These samples were incubated overnight at 4 °C with an anti-CD68 antibody (1:200), followed by incubation with secondary antibodies for 1 h at room temperature. After staining the nucleus with hematoxylin for 5 min, the images were viewed under a Nikon Eclipse E600 microscopy system (400×; Nikon Instruments, Melville, NY, USA). The Ras scores were determined using quantitative image analysis software (Pax-it, Paxcam; Villa Park, IL, USA).

### 4.7. Western Blot Analysis

Immunoblot analysis was performed according to the standard protocols [36]. After resolution on the sodium dodecyl sulfate polyacrylamide gel electrophoresis gels, the proteins were electroblotted onto nitrocellulose membranes that were subsequently blocked with 1% (*w/v*) non-fat milk in Tris-buffered saline containing 0.1% (*v*/*v*) Tween-20 (TBST). The membranes were washed with TBST and incubated with the indicated primary antibody in TBST. After extensive washing with TBST, the blot was incubated with the appropriate horseradish peroxidase-conjugated secondary antibody. The band detection was conducted using enhanced chemiluminescence (ECL) Western blotting detection reagents and exposure to ECL Hyperfilm on a FUJIFILM Las-3000 system (FUJFILM, Tokyo, Japan). The proteins were then quantitatively determined using densitometry with FUJFILM-Multi Gauge v2.2 software (FUJFILM, Tokyo, Japan).

### 4.8. Statistical Analysis

All the statistical analyzes were performed using Sigma Stat software (v3.5), and the descriptive statistics are presented as the mean ± standard deviation (SD). The between-group differences were analyzed using Student’s *t*-test. Moreover, *p* < 0.05 was considered to be statistically significant [37].

## 5. Conclusions

In the model of db/db mice fed with HFD, although Ab did not lower the blood sugar, it prevented DN via regulating the Ras, reducing renal fibrosis, and improving the renal function. 

## Figures and Tables

**Table 1:** The plasma biochemical parameters in HFD-fed mice treated with acarbose.

	Control	db/db	db/db + HFD	db/db + HFD +Ab
HbA1c(%)	4.00 ± 0.10	8.90 ± 0.57 ^#^	9.30 ± 0.56 ^#^	8.03 ± 1.03
Insulin (pg/L)	1.02 ± 0.03	5.1 ± 0.12 ^#^	7.97 ± 0.54 ^#^	5.40 ± 0.33 *
HOMA-IR	0.25 ± 0.02	5.55 ± 0.29 ^#^	11.6 ± 0.30 ^#^	7.44 ± 0.25 *
Cholesterol (mg/dL)	78.33 ± 7.22	219.00 ± 4.51 ^#^	450.00 ± 18.52 ^#^	335.00 ± 22.85 *
TG (mg/dL)	86.67 ± 2.73	150.33 ± 2.67 ^#^	357.67 ± 18.77 ^#^	175.33 ± 18.70 *
HDL-C (mg/dL)	46.00 ± 4.73	114.67 ± 1.67 ^#^	144.00 ± 5.86 ^#^	119.33 ± 7.26 *
LDL-C (mg/dL)	15.33 ± 2.03	23.00 ± 5.51 ^#^	167.00 ± 2.65 ^#^	66.33 ± 11.26 *
Cholesterol/HDL-C	1.70	1.91	3.13	2.81
BUN (mg/dL)	16.40 ± 1.72	22.90 ± 0.47 ^#^	30.07 ± 0.38 ^#^	20.10 ± 0.91 *
CRE (mg/dL)	0.43 ± 0.03	0.63 ± 0.03 ^#^	0.87 ± 0.03 ^#^	0.60 ± 0.06 *
UA (mg/dL)	3.07 ± 0.09	5.03 ± 0.19 ^#^	9.87 ± 0.19	4.93 ± 0.20 *

**Control**, C57BL/6 mice were fed with normal diet group; **db/db**, db/db mice were fed with normal diet group; **db/db+HFD**, db/db mice were fed with HFD group; **db/db+HFD+Ab**, db/db mice were fed with HFD and acarbose group. HbA1c, glycated hemoglobin. HOMA-IR, homeostasis model assessment for insulin resistance. TG, triglyceride. HDL-C, high density lipoprotein-cholesterol. LDL-C, low density lipoprotein-cholesterol. BUN, blood urea nitrogen. CRE, creatinine. UA, uric acid. Each value is expressed as the mean ± SD (*n* = 3/group). Results were statistically analyzed with Student’s *t*-test. #, *p* < 0.05 compared with the control group. *, *p* < 0.05 compared with db/db+HFD group.

## Data Availability

Data are contained within the article.
